# The Influence of Workplace-Integrated Exercise Snacks on Cognitive Performance in Sedentary Middle-Aged Adults—A Randomized Pilot Study

**DOI:** 10.3390/sports13060186

**Published:** 2025-06-13

**Authors:** Jonas P. Mues, Stefan Flohr, Nicolas Kurpiers

**Affiliations:** Department of Sports Science, University of Hildesheim, Universitätsplatz 1, 31141 Hildesheim, Germany; flohrs@uni-hildesheim.de (S.F.); kurpiers@uni-hildesheim.de (N.K.)

**Keywords:** cognitive performance, exercise snacks, motion stimulus, workplace health promotion

## Abstract

Sedentary behavior is increasingly recognized as a risk factor for various health issues, including cognitive decline. Objectives: This pilot study examined the acute and chronic effects of workplace-integrated exercise snacks—short, vigorous bouts of exercise—on cognitive performance in middle-aged adults. Methods: Twenty-five sedentary but healthy office workers (51.4 ± 5.3 years; 6 m/19 f) participated. The intervention group (*n* = 12) performed three 1 min bouts of vigorous exercise (running on the spot) daily, four days a week, for four weeks, while the control group (*n* = 13) maintained their usual routine. Cognitive performance was assessed pre-intervention, shortly following the first exercise bout (acute effects), and post-intervention (chronic effects) using the Trail Making Test and the Stroop Color–Word Test. Results: Significant acute improvements (*p* ≤ 0.05) were observed across all cognitive outcomes following a single bout of exercise. Significant interaction effects (*p* ≤ 0.05) were found across all cognitive outcomes after four weeks, indicating sustained cognitive benefits. Conclusion: These findings suggest that workplace-integrated brief, vigorous exercise may lead to both immediate and sustained enhancement in executive functions such as working memory, processing speed, and inhibitory control. They highlight the potential cognitive health benefits of incorporating exercise snacks into sedentary workplace environments.

## 1. Introduction

With the development of technology and an ongoing trend toward digital work environments, sedentary occupations with prolonged sitting times and inactive lifestyles have become increasingly prevalent. Sedentary behavior is defined as any waking behavior characterized by an energy expenditure of 1.5 metabolic equivalents of task (MET) or less, involving sitting, reclining, or lying down [1], whereas physical inactivity is defined as the failure to meet physical activity guidelines established by the WHO of at least 150 min of moderate or 75 min of vigorous aerobic physical activity per week [2]. A distinction must be made between these conditions, as they constitute independent risk factors for health-related issues [1,3]. Even individuals who meet general activity guidelines but engage in prolonged sedentary behavior throughout the rest of the day are at a significantly greater risk of adverse effects on physiological and psychological health and performance [4,5]. They show an increase in cardiometabolic risk markers, a higher incidence of type 2 diabetes, obesity, and all-cause mortality [6,7,8,9]. Moreover, sedentary behavior also has adverse effects on psychological and neurological parameters. This includes, for example, a higher prevalence of depression and an increase in dementia risk [6,10,11,12].

Recent studies have reported a negative correlation between sitting time and cognitive performance (CP), with a decline in CP with increasing daily sitting time [13]. However, CP is an important basis for different aspects of life, such as academic success [14,15,16], mental well-being [17,18], and social development [19]. Considerable evidence supports the positive effects of physical exercise [20,21] and breaking up prolonged sitting [8,22] on CP. However, while the benefits of an active lifestyle are widely recognized, many individuals still fail to integrate physical activity into their daily life and work routine because of perceived barriers such as time constraints, limited access to equipment or training facilities, and low self-efficacy [23]. Therefore, finding ways to integrate physical activity and breaks in sitting time into the daily life of those individuals at risk is highly important.

High-intensity interval training and sprint interval training have proven to be time-efficient and effective training methods for improving acute and long-term health and performance [24,25,26,27]. Short physical activities with high intensity tend to have better effects on CP than longer moderate exercise does [28,29]. Although highly efficient and effective, the high level of physical exertion associated with these training protocols makes them challenging to incorporate into the daily work routine for most people. Recent findings have shown positive effects of interval training in adults when the training intervals are not performed en bloc but are spread throughout the day in the form of very short, intense bouts of physical exercise. These so-called exercise snacks (ESs) have been shown to improve cardiorespiratory fitness and cardiovascular and metabolic risk factors in clinical and preclinical populations [30,31,32] and reduce the prevalence of cancer [33], and could constitute an effective and feasible strategy for incorporating regular physical activity into daily work life. However, the data concerning the effects of ESs are still limited, particularly with respect to mental and cognitive effects [34]. To date, only two studies have examined the potential effects of an ES intervention on CP. Stenling et al. (2019) documented positive effects on mood and CP after an acute bout of ESs (3 × 1 min stair-climbing) among healthy young adult men under laboratory conditions [35]. Nasrollahi et al. (2022) reported similar effects following a single 6 × 1 min stair-climbing session in older adults of both sexes [36]. While these studies imply the potential of ESs for enhancing cognitive function, data on the acute and chronic effects of ESs on CP in middle-aged adults in real-life settings are lacking.

The aim of the present study was (1) to investigate short-term (acute) effects of a single workplace-integrated ESs consisting of one minute of vigorous exercise and (2) to investigate long-term (chronic) effects of workplace-integrated ESs conducted three times per day on four days a week over a four-week period, totaling 48 sessions, on the CP of middle-aged adult individuals exhibiting sedentary working behavior. To the best of our knowledge, this is the first study to investigate the effect of ESs on CP under real-life conditions and aims to provide preliminary insights into their potential impact. The following exploratory hypotheses were formulated: (1) a single bout of workplace-integrated ESs may lead to acute improvements in CP compared to baseline, and (2) engaging in regular workplace-integrated ESs over a four-week period may lead to greater post-intervention improvements in CP compared to a non-intervention control group.

## 2. Methods

### 2.1. Study Design

**Figure 1:**
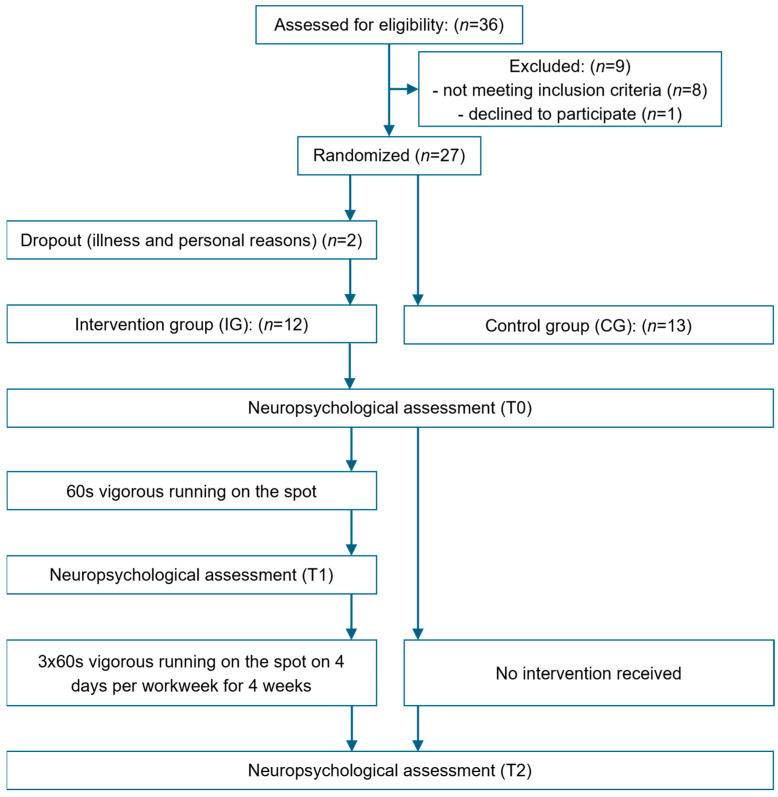
Overview of the study design.

A randomized pre- and post-test design was employed to evaluate (1) acute effects of a single bout of 60 s of running on the spot on CP within the intervention group (IG) and (2) the chronic effects of 4 weeks of ESs, comprising 60 s of running on the spot, 3 times a day for 4 days per week of the IG, in comparison to a control group (CG) receiving no intervention (Figure 1). Depending on group allocation, participants underwent either two (CG) or three (IG) neuropsychological assessments. On the initial testing day (T0), participants in both groups completed the neuropsychological assessment to establish baseline values. In the IG, the 4-week intervention period commenced one week after T0. On the first day of the intervention period, participants in the IG completed the second neuropsychological assessment (T1) following a five-minute rest after the first ES to evaluate acute effects on CP [37]. Three days after the end of the 4-week intervention period, all participants underwent a final neuropsychological assessment (T2) to measure post-test values (chronic effect). Participants were instructed not to engage in physical activity prior to the final assessment to ensure that there was no interference from acute activation following physical exercise.

### 2.2. Participants

Prior to study enrollment, participants provided signed privacy declarations and informed written consent forms. This study was approved by the Ethics Committee of the University of Hildesheim. All participants were recruited from a local bank branch, i.e., employees with a high amount of daily sedentary work time. The recruitment period spanned from 8 January 2024 to 5 February 2024. The inclusion criteria were an age between 30 and 60 years; the absence of neurological, orthopedic, or cardiovascular diseases (assessed via self-reports); eight or more hours per day of sedentary behavior (assessed via self-reports); and readiness to engage in physical activity (assessed via the Get Active Questionnaire; GAQ [38]). The exclusion criteria were color blindness, a positive response to any question on the GAQ form, and the interruption of the intervention for more than 3 days. A total of 27 middle-aged adults initially participated in this study. Two participants from the IG dropped out during the first week of the intervention due to illness and personal reasons and were not included in the analysis. As a result, a total of 25 participants (6 males, 19 females; mean age 51.4 ± 5.3 years; daily sedentary time 9.9 ± 1.9 h) completed the study protocol. An overview of the general characteristics of each group is shown in Table 1.

### 2.3. Randomization

To prevent unequal assignments of confounding age groups, the cohort was stratified by age. Participants were then randomly assigned to either the intervention group or the control group via block randomization. A combination of six blocks of 4 and one block of 3 was used to accommodate all 27 participants. All possible balanced combinations for blocks of 4 (2 × 2) and blocks of 3 (2 × 1 or vice versa) were determined. From these, six randomly selected block combinations (block size of 4) and one randomly selected combination (block size of 3) determined the final assignment. This process resulted in two balanced groups: 14 participants in the intervention group (4 males/10 females, mean age: 51.3 ± 5.5) and 13 in the control group (2 males, 11 females, mean age: 52.2 ± 5.1).

### 2.4. Intervention

The IG participants followed an ES protocol adopted from Jenkins et al. (2019) and Islam et al. (2022) [31,32]. The protocol demanded three bouts of 60 s of vigorous exercise (running on the spot) on 4 days per workweek (Monday–Friday), adding up to 48 exercise sessions in total. The time span between two ES sessions should be at least one hour and not exceed four hours. To assess training intensity and perceived effort, participants recorded their rating of perceived exertion (RPE) via the Borg category ratio scale (CR10) immediately after each exercise bout. The CR10 scale was used because of its reliability in monitoring office exercise training intensity [39]. Whenever feasible, ES bouts were scheduled during work, although this was not always possible, e.g., if participants were employed part-time. Proper and safe movement execution was checked individually before the start of the intervention and corrected if necessary.

### 2.5. Neuropsychological Assessment

The Trail Making Test (TMT) [40] was utilized to measure CP. The TMT is widely used internationally in neuropsychological screening and is highly sensitive in assessing executive function in adults [41,42,43]. The test comprises two parts (TMT-A and TMT-B), requiring participants to connect encircled numbers (1–25) in ascending order (TMT-A) and encircled numbers (1–13) and letters (A–L) in alternating order (TMT-B) as quickly as possible via pencil lines on a semi-randomly arranged DIN-A4 sheet. The test is scored using a time-based metric, with lower scores indicating better performance. Studies indicate that both parts of the test involve graphomotor speed and visual search, with TMT-B additionally assessing higher-level executive functions such as cognitive flexibility and working memory [43,44,45]. Two alternate, mirrored forms of the TMT were used for the assessment of acute (TMT-A_1_ and TMT-B_1_) and chronic effects (TMT-A_2_ and TMT-B_2_) to minimize learning effects, following recommendations by Wagner et al. (2011) [46]. These alternate forms have demonstrated high test–retest reliabilities ranging from *r* = 0.76 to 0.89 for TMT-A and *r* = 0.86 to 0.94 for TMT-B. Test administration followed the protocol described by Strauss et al. (2006) [41], with instructions translated into the German language.

The Stroop Color–Word Test (SCWT) [47] was employed to assess executive functioning, specifically inhibitory control, selective attention, cognitive flexibility, and processing speed [48,49,50]. The standardized version by Golden (1978) was adopted for its extensive normative data and widespread use in the literature [41]. The test consists of three parts: (I) word-reading (W), (II) color-naming (C), and (III) color–word (C/W). Each part of the test assesses different cognitive constructs, with word-reading (W) primarily measuring processing speed, color-naming (C) reflecting working memory and visual search speed, and color–word (C/W) evaluating processing speed, working memory, and inhibitory control [51]. Each part comprises a white DIN-A4 sheet with 100 items arranged in a 5 × 20 matrix, requiring participants to correctly name as many items as possible within a 45 s timeframe. The test is scored based on the number of correctly named items within the time limit, with higher scores indicating better performance. The reported test–retest reliabilities are *r* = 0.89 for W, *r* = 0.84 for C, and *r* = 0.73 for C/W [41]. In Part I, color–words (red, blue, and green) are printed in black ink with no words allowed to follow themselves. Part II consists of colors (written as XXXX) printed in red, blue, or green, with no color allowed to follow itself. Part III consists of the same color–words as in part I, printed in the colors used in part II to ensure that no color–word matches the corresponding color of the ink.

### 2.6. Standardization

Neuropsychological assessments were conducted at the same time of day (±120 min), as the outcome of the Stroop Color–Word Test varies depending on the time of day when administered to older adults [41]. The participants were instructed to consume a typical breakfast on the day of the trial and abstain from alcohol on the day preceding each trial, as confirmed through self-reports. Additionally, participants were prohibited from making changes to their daily routine, such as nutrition, physical activity level, or sleep schedule, unless necessary for this study [52]. Finally, the participants were instructed to refrain from practicing the neuropsychological tests to mitigate the potential impact of practice effects.

### 2.7. Data Analysis

The statistical analysis was conducted via IBM SPSS Statistics Software (version 29.0.0.0; IBM Corp., Armonk, NY, USA). Given a sample size of ≤50, the normality of distribution of the data was tested via the Shapiro–Wilk test. The homogeneity of variances was proven by using Levene’s test. Statistical significance was set at *p* ≤ 0.05. The acute effect, i.e., the results obtained from T0 and T1, was analyzed using paired-samples *t*-tests and Hedges’s *g*. The effect size was rated as follows: 0.2 (small effect), 0.5 (medium effect), and 0.8 (large effect) [53]. A 2 × 2 mixed-design ANOVA with time (T0, T2) as a within-subject factor and group (IG, CG) as a between-subject factor was used to analyze the chronic effect of the intervention. Partial eta squared tests were reported as a measure of effect size and rated as follows: 0.01 (small effect), 0.06 (medium effect), and 0.14 (large effect) [53].

## 3. Results

### 3.1. Acute Effects

All neuropsychological test scores showed improved mean values from T0 to T1 (lower values in TMT-A and TMT-B; higher values in SW, SC, and SCW; see Table 2). Paired-samples *t*-tests revealed significant differences across all measures of CP. For the Trail Making Test, significant differences were found for Part A (*t* (11) = 3.16, *p* = 0.005, *g* = 0.85) and for Part B (*t* (11) = 2.39, *p* = 0.018, *g* = 0.64). For the Stroop Color–Word Test, significant differences were found for SW (*t* (11) = −7.60, *p* < 0.001, *g* = −2.14), SC (*t* (11) = −3.17, *p* = 0.004, *g* = −0.85) and SCW (*t* (11) = −4.37, *p* < 0.001, *g* = −1.17).

### 3.2. Chronic Effects

**Figure 2:**
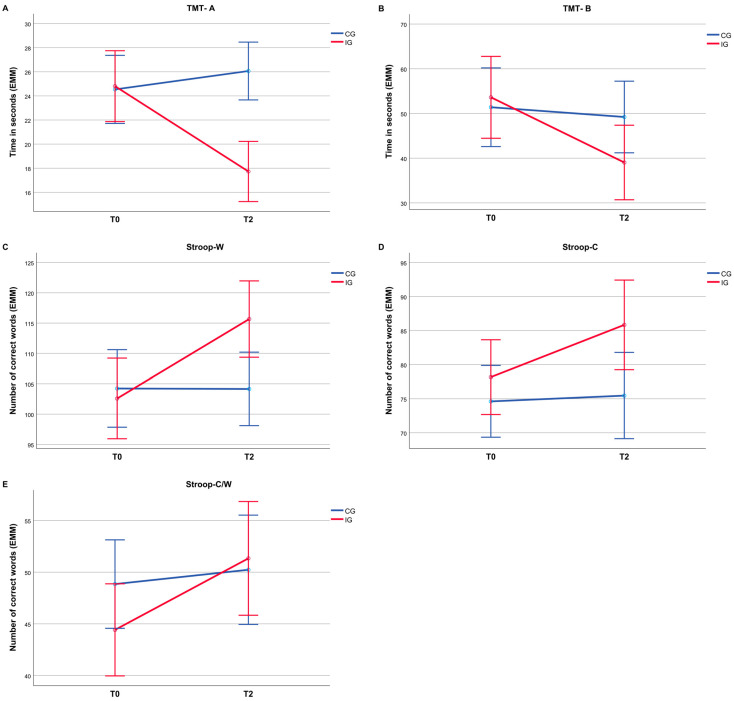
Estimated marginal means (EMMs) for all measures of cognitive performance for CG and IG at T0 and T2. (**A**) Trail Making Test Part A; (**B**) Trail Making Test Part B; (**C**) Stroop Word; (**D**) Stroop Color; (**E**) Stroop Color-Word. Error bars indicate ±2 SE.

A descriptive overview of the neuropsychological assessment results for the pretest (T0) and post-test (T2) is shown in Table 3. The 2 × 2 mixed-design ANOVA revealed significant interaction effects for all cognitive outcomes, as shown in Table 4. For the Trail Making Test, significant interactions were found for both Part A (*F*(1, 23) = 19.80, *p* < 0.001, $\eta_{p}^{2}$ = 0.46; Figure 2A) and Part B (*F*(1, 23) = 6.18, *p* = 0.021, $\eta_{p}^{2}$ = 0.21; Figure 2B). For the Stroop Color–Word Test, significant interaction effects were found for SW (*F*(1, 23) = 28.51, *p* < 0.001, $\eta_{p}^{2}$ = 0.55; Figure 2C), SC (*F*(1, 23) = 11.20, *p* = 0.003, $\eta_{p}^{2}$ = 0.33; Figure 2D), and SCW (*F*(1, 23) = 13.64, *p* = 0.001, $\eta_{p}^{2}$ = 0.37; Figure 2E).

## 4. Discussion

The present study was the first to investigate the acute and chronic effects of brief bouts of vigorous exercise dispersed throughout a workday on CP among sedentary healthy adults. This is an important direction for future research owing to our limited understanding of how exercise intensity, duration, and volume affect CP in sedentary clinical and preclinical populations.

This study revealed a significant acute enhancement in the performance of neuropsychological tests (TMT and SCWT) following a short, vigorous bout of exercise (running on the spot) under real-life conditions after the first ES (T1 compared with T0). The increase in TMT performance suggests an improvement in executive function, such as graphomotor speed, visual search, cognitive flexibility, and working memory, with potentially considerable practical significance highlighted by the large effect size revealed in the analysis. These findings are supported by the SCWT results, further indicating substantial acute benefits for cognitive domains such as processing speed and inhibitory control among middle-aged adults. Overall, these study results are generally consistent with the current literature on the positive effects of physical exercise on cognitive function, especially regarding the TMT and SCWT, in adults [20,21,37,54]. The effects observed in this study differ from those reported in large meta-analyses, which have consistently reported only small positive effects of acute exercise on cognitive function [37,55]. This discrepancy could result from the greater intensity at which the exercise was performed in this study than in most studies included in the meta-analyses. This proposition is supported by research showing a correlation between exercise intensity and CP, favoring high intensities [21,22,28,29,56].

Drawing upon the concept of ESs, Stenling et al. (2019) reported acute effects on switching performance among active young adult males but not females. Moreover, no differences in enhanced inhibitory control were observed across participants [35]. The results of the present study differ from these findings, and several potential explanations must be considered. First, there were differences in participants across the studies. While Stenling et al. (2019) recruited young adults (mean age of 19.4 years) for their study, the cohort of the present study was considerably older (mean age of 51.7 years) and generally characterized as more sedentary. Given that increased age [41,57] and sedentary behavior [13] have been identified as negative correlates of CP, one might expect that the cohort in this study would be more predisposed to improvements in CP than the study conducted by Stenling et al. (2019). This explanation is supported by another investigation by Nasrollahi et al. (2022), who adopted the study design proposed by Stenling et al. (2019) for older adults and discovered enhancements in all measures of CP for both sexes [36]. Additionally, there was a discrepancy in the choice of tests used to measure inhibitory control between studies. As Stenling et al. (2019) noted, the Stroop Color–Word Test utilized in this study might be more sensitive in measuring the effects of short, vigorous exercises on inhibitory control than the test employed in their study [35]. Overall, the exact underlying mechanism of the effects of acute exercise on CP remains unclear. Recent studies suggest that acute intense exercise promotes positive effects on CP as a consequence of an increase in the release of brain-derived neurotrophic factors (BDNFs) and insulin-like growth factor-1 (IGF-1) and an increase in cerebrovascular blood flow [21,28,29,35].

The second aim of this study was to investigate the chronic effects of ES bouts dispersed throughout the workday on CP in sedentary healthy adults. This study revealed a significant enhancement in performance across all measures of neuropsychological assessment (TMT and SCWT) following the ES sessions. These findings imply a positive post-intervention effect of integrating regular short bouts of exercise on executive function into the work routine of a predominantly sedentary population prone to extended periods of uninterrupted sitting. The corresponding literature regarding this aim of this study presents an inconsistent picture. Interventions aiming to break up prolonged sitting merely by changing posture from sitting to standing have been found to have no effect on CP [8,22]. However, breaking up sitting time by incorporating physical activities such as cycling or walking has improved certain domains of CP in adults [8,22]. This suggests that a certain threshold of physical activity must be reached during breaks to achieve lasting effects on CP, favoring higher intensities of exercise over light and moderate intensities, as also suggested by Tuckwell et al. (2019) [22]. These findings are in line with the results of this study. One possible explanation could be the increase in cardiorespiratory activation resulting from increased exercise intensity [24,25,26,27]. Studies suggest a strong correlation between cardiorespiratory fitness and CP in middle-aged and older adults, particularly among those with initially low fitness levels [58,59]. In previous studies on the physical effects of 6-week ES interventions, which involved 3 bouts of 1 min of vigorous stair-climbing 3 days per week (totaling 54 bouts compared to 48 bouts in this study), significant increases in VO_2peak_ of 5% [31] and 7% [30] were observed in the intervention groups. It can therefore be expected that an increase in cardiorespiratory fitness occurred during this study within the intervention group as well, which likely contributed to the enhanced CP. However, CP is a complex construct, and there are likely a multitude of mechanisms involved that contribute to the study results. Since the investigation of the underlying mechanisms was not the focus of this study, exploring those avenues is an important future direction. The incorporation of measures of these mechanisms in future studies could provide a better understanding of the interaction between cognition and exercise.

The present study has several limitations that need consideration. Since the cohort mainly consisted of female participants and the groups were not balanced regarding sex, larger sample size studies including sex-balanced cohorts are needed in the future to elucidate potential sex-specific effects. Furthermore, while participants were instructed to refrain from changing their daily routines, such as nutrition, physical activity level, or sleep schedule, it is not possible to rule out that the participants consciously or subconsciously changed aspects of their daily life toward a healthier lifestyle, ultimately altering the test results. A limitation regarding the interpretation of acute effects (T1) is the fact that the assessment was not controlled. This procedural decision to exclude the control group from this assessment had to be made based on practical considerations during study planning.

## 5. Conclusions

This study indicates that incorporating regular three bouts of 1 min of vigorous exercise into the work routine of sedentary middle-aged adults can lead to a significant increase in acute cognitive function and post-intervention enhancements following a four-week intervention period. Finally, this study observed that “exercise snacking” is a feasible strategy in an office environment to effectively enhance CP in sedentary adults.

## Figures and Tables

**Table 1:** General characteristics of the participants.

	Intervention Group	Control Group
Participants [*n*] (m/f)	12 (4/8)	13 (2/11)
Age [years] ^1^	50.6 ± 5.6	52.2 ± 5.1
Sedentary Time [hours per day] ^1^	10.5 ± 2.3	9.5 ± 1.5

^1^ Data given as mean ± standard deviation.

**Table 2:** Descriptive and inferential statistics of cognitive performance for T0 and T1 (acute effect).

	T0 ^1^	T1 ^1^	Δ ^1^	*p* ^2^	*g*
TMT-A [s]	24.8 ± 6.4	19.6 ± 4.4	−5.2 ± 5.8	0.005 *	0.85
TMT-B [s]	53.6 ± 18.2	43.0 ± 9.7	−10.6 ± 15.5	0.018 *	0.64
SW [score]	102.6 ± 10.0	113.1 ± 10.6	10.5 ± 4.6	<0.001 *	−2.14
SC [score]	78.2 ± 8.8	82.5 ± 10.5	4.3 ± 4.7	0.004 *	−0.85
SCW [score]	44.4 ± 4.9	49.0 ± 6.8	4.6 ± 3.6	<0.001 *	−1.17

Δ = Mean difference between T1 and T0; *g* = Hedges’s *g* (effect size); TMT = Trail Making Test (lower scores indicate better performance); SW = Stroop Word (higher scores indicate better performance); SC = Stroop Color (higher scores indicate better performance); SCW = Stroop Color–Word (higher scores indicate better performance). ^1^ Data given as mean ± standard deviation. ^2^ Asterisks indicate significant differences (*p* ≤ 0.05).

**Table 3:** Descriptive statistics of cognitive performance for T0 and T2 (chronic effect).

Test	Group	T0 ^1^	T2 ^1^	Δ ^1^
TMT-A [s]	IG	24.8 ± 6.4	17.7 ± 3.3	−7.1 ± 5.0
	CG	24.5 ± 3.5	26.1 ± 5.1	1.6 ± 4.7
TMT-B [s]	IG	53.6 ± 18.2	39.0 ± 7.9	−14.6 ± 13.0
	CG	51.4 ± 13.4	49.2 ± 18.5	−2.2 ± 11.9
SW [score]	IG	102.6 ± 10.0	115.7 ± 11.8	13.1 ± 5.5
	CG	104.2 ± 12.7	104.2 ± 10.0	0.0 ± 6.7
SC [score]	IG	78.2 ± 8.8	85.8 ± 11.0	7.6 ± 5.9
	CG	74.6 ± 10.1	75.5 ± 11.8	0.9 ± 4.2
SCW [score]	IG	44.4 ± 4.9	51.3 ± 5.5	6.9 ± 3.0
	CG	48.9 ± 9.6	50.2 ± 12.1	1.3 ± 4.3

Δ = Mean difference between T2 and T0; TMT = Trail Making Test (lower scores indicate better performance), SW = Stroop Word (higher scores indicate better performance), SC = Stroop Color (higher scores indicate better performance); SCW = Stroop Color–Word (higher scores indicate better performance). ^1^ Data given as mean ± standard deviation.

**Table 4:** Inferential statistics of cognitive performance for T0 and T2 (chronic effect).

Test	*F* (Time)	*p* ^1^	$\eta_{p}^{2}$	*F* (Group)	*p* ^1^	$\eta_{p}^{2}$	*F* (Time × Group)	*p* ^1^	$\eta_{p}^{2}$
TMT-A	8.25	0.009 *	0.26	6.16	0.021 *	0.21	19.80	<0.001 *	0.46
TMT-B	11.30	0.003 *	0.33	0.52	0.479	0.02	6.18	0.021 *	0.21
SW	27.85	<0.001 *	0.55	1.30	0.265	0.05	28.51	<0.001 *	0.55
SC	17.44	<0.001 *	0.43	2.92	0.101	0.11	11.20	0.003 *	0.33
SCW	30.72	<0.001 *	0.57	0.24	0.628	0.01	13.64	0.001 *	0.37

*F*(time) = main effect of time; *F*(group) = main effect of group; *F*(time × group) = interaction effect; $\eta_{p}^{2}$ = partial eta squared (effect size); TMT = Trail Making Test; SW = Stroop Word; SC = Stroop Color; SCW = Stroop Color–Word. ^1^ Asterisks indicate significant differences (*p* ≤ 0.05).

## Data Availability

The data that support the findings of this study will be made available upon reasonable request to the corresponding author. The data are not publicly available due to containing information that could compromise the privacy of research participants.
